# Cellular Effects of Butyrate on Vascular Smooth Muscle Cells are Mediated through Disparate Actions on Dual Targets, Histone Deacetylase (HDAC) Activity and PI3K/Akt Signaling Network

**DOI:** 10.3390/ijms20122902

**Published:** 2019-06-14

**Authors:** Omana P. Mathew, Kasturi Ranganna, Joseph Mathew, Meiling Zhu, Zivar Yousefipour, Chelliah Selvam, Shirlette G. Milton

**Affiliations:** Department of Pharmaceutical and Environmental Health Sciences, College of Pharmacy and Health Sciences, Texas Southern University, 3100 Cleburne St, Houston, TX 77004, USA; Omanapmathew@yahoo.com (O.P.M.); josephmk@tsu.edu (J.M.); anniezhu2010@gmail.com (M.Z.); Yousefipour_zx@tsu.edu (Z.Y.); chelliahs@tsu.edu (C.S.); Shirlette.milton@tsu.edu (S.G.M.)

**Keywords:** vascular smooth muscle cells, butyrate, histone deacetylase inhibitor, signaling, Akt

## Abstract

Vascular remodeling is a characteristic feature of cardiovascular diseases. Altered cellular processes of vascular smooth muscle cells (VSMCs) is a crucial component in vascular remodeling. Histone deacetylase inhibitor (HDACI), butyrate, arrests VSMC proliferation and promotes cell growth. The objective of the study is to determine the mechanism of butyrate-induced VSMC growth. Using proliferating VSMCs exposed to 5 mM butyrate, immunoblotting studies are performed to determine whether PI3K/Akt pathway that regulates different cellular effects is a target of butyrate-induced VSMC growth. Butyrate inhibits phosphorylation-dependent activation of PI3K, PDK1, and Akt, eliciting differential effects on downstream targets of Akt. Along with previously reported Ser9 phosphorylation-mediated GSK3 inactivation leading to stability, increased expression and accumulation of cyclin D1, and epigenetic histone modifications, inactivation of Akt by butyrate results in: transcriptional activation of FOXO1 and FOXO3 promoting G1 arrest through p21Cip1/Waf1 and p15INK4B upregulation; inactivation of mTOR inhibiting activation of its targets p70S6K and 4E-BP1 impeding protein synthesis; inhibition of caspase 3 cleavage and downregulation of PARP preventing apoptosis. Our findings imply butyrate abrogates Akt activation, causing differential effects on Akt targets promoting convergence of cross-talk between their complimentary actions leading to VSMC growth by arresting proliferation and inhibiting apoptosis through its effect on dual targets, HDAC activity and PI3K/Akt pathway network.

## 1. Introduction

Vascular remodeling is a characteristic pathological trait of many of the cardiovascular diseases and disorders, including atherosclerosis and restenosis through the changes in cell proliferation, cell growth, apoptosis, and differentiation of vascular cells in concert with vascular homeostasis. Cardiovascular disease continues to be a major health concern in spite of great strides that have been made in understanding the pathogenesis and management of the disease [1,2,3]. This suggests that besides the known causes and associated risk factors, there may be other mechanisms playing a role in cardiovascular events. Recent upsurge in epigenetic research discloses that epigenetic processes are normal events that are vital to normal development and functioning of organisms [2,4,5], and also instigated by many factors including environmental agents, lifestyle habits, dietary factors, aging and behavior [2,4,5,6]. Epigenetic processes mediated through histone modifications, DNA methylation and microRNAs impose an added level of regulation by modulating expression of critical genes that influence several cellular effects which are important to normal physiology [2,4,5,7,8,9,10,11]. Dysregulation of epigenetic processes has been linked to several diseases especially in cancer pathogenesis [4,12,13,14,15,16]. Cardiovascular epigenetics is a relatively unexplored area of biomedical research. Epigenetic components in cardiovascular disease pathways, including the pathways linked to atherosclerosis, are now being investigated to understand their role in the pathogenesis [17,18,19]. Understanding the significance of dysregulated epigenetic changes in the development of disease offers an effective therapeutic strategy for different diseases including atherosclerosis- and restenosis-associated vessel remodeling.

Histones are targets of several different transient and reversible site-specific covalent post-translational modifications (PTMs) such as acetylation, methylation, phosphorylation, sumoylation and ADP-ribosylation. Reversible modifications of histones by acetylation alter chromatin structure, dynamics, promoter accessibility and recruitment of histone modifiers to promote changes in gene functions by affecting gene expressions [5,20,21,22]. Histone acetyltransferases (HATs) and histone deacetylases (HDACs) are responsible for writing and erasing acetylation marks on lysine residues of histone N-terminal tails, and more than 50 different non-histone proteins [21]. Counterbalancing activities of these enzymes are crucial to the regulation of gene expressions by facilitating essential processes, including transcription, replication and repair processes [5,20,21]. Importantly, inhibition of HDACs activity has been exploited in reactivating transcriptionally silent tumor suppressor genes such as p21Cip1/WAF1 to arrest cancer cell proliferation and growth by promoting histone acetylation [22,23,24,25]. This paved the way to the development of HDAC inhibitors (HDACIs) as a new class of anti-cancer drugs consisting of diverse sets of compounds, including both natural and synthetically produced compounds varying in structure, specificity and biological activity [21,22]. Several of these inhibitors have been used in clinical trials of different diseases [2,4,16,21,26]. Moreover, HDACIs cause an array of cell-type specific cellular effects in vitro and in vivo, including inhibition of cell proliferation, stimulation of cell differentiation and/or apoptosis, and migration in non-cancer and cancer cells [2,21,22,23,24,25,27,28,29,30,31,32,33,34,35,36,37].

Butyrate, a fermentation product of dietary fiber and a long known natural HDACI, also causes a variety of cellular effects such as cell-cycle arrest in G1-phase, change in cellular morphology and size, stimulate cell differentiation and/or induce apoptosis in different non-cancerous and cancerous cells [21,22,23,24,25,27,28,29,30,31,32,33,34,35,36,37]. Our earlier studies [23,24,34,36] and studies from other investigators have shown that butyrate arrests vascular smooth muscle cell (VSMC) proliferation and causes antimigratory effects, the crucial factors in the pathogenesis of vascular proliferative diseases that include atherosclerosis, arterial restenosis and hypertension [2,3,37,38,39,40]. In addition to antiproliferative and antimigratory effects, butyrate also causes anti-oxidant and anti-inflammatory effects displaying potential antiatherogenic chemopreventive and chemotherapeutic properties [41]. Furthermore, our studies have shown that butyrate arrests proliferating VSMCs in G1-phase by downregulating cyclin-dependent kinases 4 (cdk4), cdk6 and cdk2 and upregulating Cip/Kip and INK families of cdk inhibitors. Consequently, this prevents entry into S-phase due to inhibition of phosphorylation of retinoblastoma protein (Rb) [24]. Interestingly, butyrate treatment of proliferating VSMCs causes upregulation of cyclin D1 and alters cellular morphology and size promoting VSMC growth [23,24,36], unlike trichostatin A, another HDACI [36]. Our earlier study suggests that stabilization of cyclin D1 due to inhibition of cyclin D1 degradation by inactive glycogen synthase kinase 3(GSK3) combined with increase in cyclin D1 synthesis contribute to increased cyclin D1 level in butyrate-treated VSMCs. However, the underlying molecular mechanisms accountable for butyrate’s unusual effect on cyclin D1 level and altered cellular morphology of VSMCs was not clear. This is due to the realization that GSK3 may have a role in butyrate-induced VSMC effects [24,36] compounded with the knowledge that HATs and HDACs can modulate several non-histone substrates, that include signal transduction proteins and transcription factors by acetylation and deacetylation besides affecting gene expression [42,43]. In addition, it is possible that cellular effects of butyrate on VSMCs may also involve changes in signaling pathways. Moreover, phosphatidylinositol-3-kinase (PI3K)/protein kinase B (Akt) signaling pathway and downstream targets of Akt have been shown to play a central role in VSMC biology by regulating VSMC proliferation and contractility [44,45].

The PI3K/Akt pathway is central to cellular processes such as cell proliferation, growth, differentiation and cell death. Several studies have shown that inhibition of Akt activation is involved in butyrate-inhibited cell proliferation leading to cell differentiation or cell death. However, not much is known regarding the role of butyrate-inhibited Akt activation in cell growth. Therefore, the focus of our present study is to determine whether butyrate-inhibited proliferation and stimulation of growth (increase in size and mass, not in number) of VSMCs is linked to its role as a dual inhibitor of PI3K/Akt pathway and HDAC activity. Results of our study demonstrate that butyrate inhibits activation of PI3K/Akt signaling and differently alters downstream targets of Akt that appear to support cell-cycle arrest and promote cell growth by changing morphology and size of VSMCs. This action apparently affects dual targets: histone deacetylase [24,36], activity that alters gene expression via histone modification, and PI3K/Akt signaling that modulates signaling cascade through activation/inactivation of target proteins.

## 2. Results

### 2.1. Butyrate Promotes VSMC Growth by Altering Cellular Size and Morphology, and Arresting VSMC Proliferation

**Figure 1:**
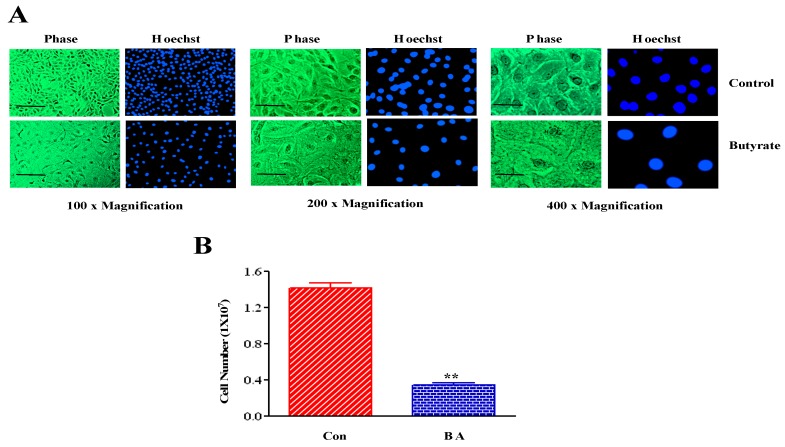
Cellular effects of butyrate on VSMCs. Proliferating VSMCs treated with (BA) or without (Con) 5 mM butyrate for 72 h and processed for assessing butyrate-induced cellular effects. (**A**) Pseudocolored phase contrast images and corresponding nuclear images of VSMCs stained with Hoechst reveal butyrate-induced altered cell morphology and increase in size. The scale bar is 50 μm. (**B**) VSMC proliferation measured by cell counting indicate proliferation arrest by butyrate. ** *p* < 0.001 vs Control (Con).

Treatment of VSMCs with butyrate for 72 h alters cellular morphology of VSMC from an elongated bipolar morphology to a round spread-out phenotype with increase in cellular size (Figure 1A). The extent of butyrate-induced increase in cellular size of VSMCs depends on the initial density of the culture. Reduction in VSMCs in butyrate-treated VSMC cultures was due to inhibition of proliferation, and not because of cell death which was confirmed by trypan blue exclusion method (Figure 1B). Butyrate treatment causes no cytotoxicity.

### 2.2. Effect of Butyrate on Phosphatidylinositol-3-Kinase (PI3K) in Proliferation Arrested VSMCs

**Figure 2:**
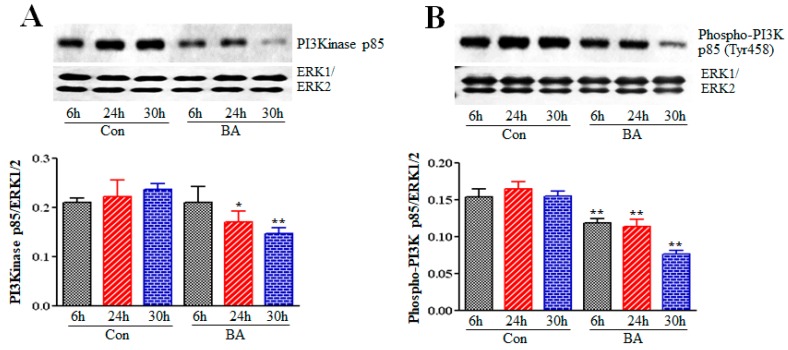
Effect of butyrate (BA) on PI3K in VSMCs. Proliferating VSMCs were treated with or without 5 mM butyrate for indicated periods of time. At the end of treatment, cell lysates were prepared and subjected to Western blot analysis to determine the PI3K expression and activation level by measuring the level of unphosphorylated and phosphorylated p85 subunit of PI3K, respectively. Immunoblotting of ERK1/2 was performed with the same lysate to normalize the protein loading. The band intensities were measured and normalized to protein loading. The data obtained were analyzed and presented as mean ± S.D. (**A**) Expression level of PI3K p85 subunit measured by using antibody specific to p85 subunit (* *p* < 0.01 vs 24 h control (Con), and ** *p* < 0.001 vs 30 h control (Con). (**B**) Activation of p85 subunit of PI3K was evaluated by the antibody specific to Tyr458-phosphorylated p85 (** *p*< 0.001 vs respective control (Con).

In response to various factors and conditions, cells are stimulated to produce second messenger phosphatidylinositol-3,4,5-triphosphate (PIP3) by activating PI3K. The important role of PIP3 is to trigger activation of signaling components that regulate proliferation, growth and survival, particularly Akt. A number of proteins having pleckstrin homology (PH) domains have strong affinity to PIP3. Since PIP3 is restricted to plasma membrane, PIP3 formation by the activation of PI3K plays an important role in the recruitment of PH domain-containing signaling proteins. These proteins include phosphoinositide–dependent protein kinase 1 (PDK1) and Akt that translocate from the cytoplasm to the plasma membrane to mediate proliferation, growth and survival signals [44,45,46,47]. To determine whether butyrate-inhibited VSMC proliferation is linked to inhibition of PI3K, the effect of butyrate on expression and activation state of PI3K is assessed by Western blotting of p85 using anti-p85 and anti-phospho-Tyr458p85 antibodies, respectively (Cell Signaling Technologies, Burlingame, CA). The data shown in Figure 2 reveals that butyrate treatment inhibits both p85 expression (Figure 2A) and p85 phosphorylation (Figure 2B) in a time-dependent manner. This indicates butyrate treatment causes downregulation of PIP3 production, the second messenger that is required for the recruitment of cytoplasmic Akt and PDK1 to the plasma membrane to mediate the proliferation signals.

### 2.3. Influence of Butyrate Treatment of VSMCs on PDK1

**Figure 3:**
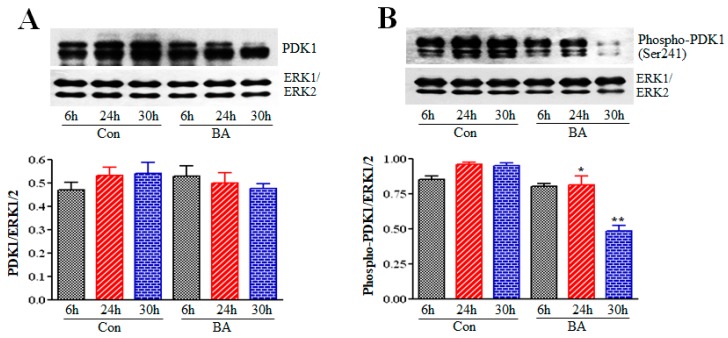
Effect of butyrate on PDK1 protein and phosphorylation level in VSMCs. Proliferating VSMCs were treated with (BA) or without (Con) butyrate for indicated periods of time and then cell lysates were prepared. Cell lysates were processed for PDK1 Western blotting to assess the effect of butyrate-inhibited PI3K activity on PDK1 protein and activation levels by measuring total and phosphorylated PDK1 levels, respectively. The data obtained were analyzed and presented as mean ± S.D. (**A**) PDK1 protein level measured by using antibody specific to total PDK1 (**B**) Phosphorylation of PDK1 assessed by using antibody specific to Ser241-phosphorylated PDK1 indicated significant reduction in activation of PDK1 in VSMCs treated with butyrate for 24 h and 30 h (* *p* < 0.01 vs 24 h control (Con), ** *p* < 0.001 vs 30 h control (Con).

PDK1 is a serine/threonine kinase and is an essential upstream activator of Akt. Activation of PI3K is necessary for the activation of PDK1, a member of the AGC superfamily of protein kinases, at the membrane to activate Akt, where it binds to PIP3 on plasma membrane formed by PI3K catalytic activity through its PH domain [44,45,46,47,48]. To evaluate the influence of butyrate-inhibited PI3K on activation of PDK1 at the plasma membrane, PDK1 expression and activation state was investigated using anti-PDK1 and anti-phospho-Ser241PDK1 antibodies, respectively (Cell Signaling Technologies, Burlingame, CA). Although butyrate treatment of VSMCs causes slight reduction in PDK1 protein levels (Figure 3A), it inhibits phosphorylation of PDK1 significantly in a time-dependent manner (Figure 3B) indicating reduction in PI3K activation by butyrate correspondingly provokes inhibition of activation of PDK1.

### 2.4. Butyrate Treatment of VSMCs Impedes Activation of Akt

**Figure 4:**
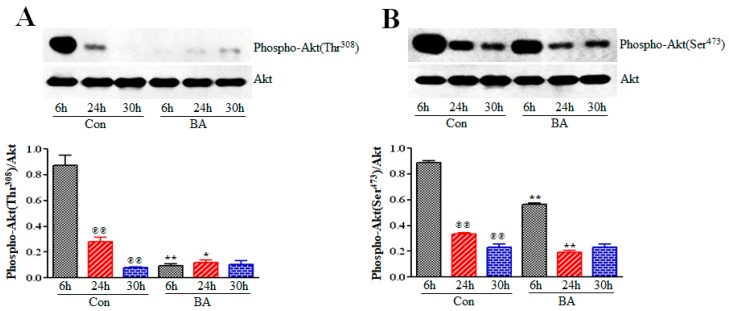
Inhibition of Akt activation by butyrate. VSMCs were treated with (BA) or without (Con) butyrate for indicated periods of time and then processed for western analysis of total Akt, Thr308 and Ser473 phosphorylated Akt as described in Methods Section. Total Akt levels were determined by immunoblotting with Akt-specific antibody and used as normalizing controls. Respective data are presented as mean ± S.D. (**A**) Levels of Thr308-phosphorylated Akt were determined by immunoblotting with antibody specific to phospho-Thr308Akt. (**B**) Levels of Ser473-phosphorylated Akt were assessed by immunoblotting with antibody specific to phospho-Ser473Akt. ^℗℗^ *p* < 0.001 vs 6 h control (Con); ** *p*< 0.001 vs 6 h control (Con), * *p* < 0.01 vs 24 h control (Con).

Akt, a serine/threonine kinase that is also referred to as protein kinase B (PKB), regulates several cellular effects including, cell proliferation, cell growth, survival, cell death and migration by regulating several downstream signaling effector proteins [44,45,46,47]. Akt is inactive and resides in the cytoplasm until the cells are stimulated. On stimulation, Akt translocates to the plasma membrane and interacts with PIP3 through its PH domain, promoting its conformational change and exposing phosphorylation sites Thr308 and Ser473 required for its activation. PDK1 catalyzes phosphorylation of Thr308 site causing partial activation of Akt. However, phosphorylation of Ser473 is essential for the full activation of Akt, and several different kinases can catalyze the phosphorylation of Ser473, including mammalian-target of rapamycin complex 2 (mTORC2). Since activation of both PI3 K and PDK1 is downregulated in butyrate-treated VSMCs, it is expected that Akt activation is compromised and blocks signals necessary for VSMC proliferation. To infer whether Akt activation is compromised in butyrate-arrested VSMC proliferation, its influence on Akt is determined by Western blotting using anti-Akt, anti-phospho-Thr308Akt and anti-phospho-Ser473Akt antibodies specific to total Akt, Thr308 and Ser473 phosphorylated Akt, respectively. As shown in Figure 4, phosphorylation of Thr308 (Figure 4A) and Ser473 (Figure 4B) residues of Akt increased in 6 h untreated VSMCs, but their phosphorylation was significantly reduced in 24 h and 30 h untreated VSMCs, with no effect on total Akt. Conversely, phosphorylation of Thr308 (Figure 4A) and Ser473 (Figure 4B) residues of Akt was strongly inhibited in 6 h and 24 h butyrate-treated VSMCs, and in 30 h treated VSMCs, slight reversal of butyrate-inhibited Thr307 and Ser473 phosphorylation was observed.

### 2.5. Role of Downstream Targets of Akt in Butyrate-Induced Cellular Effects of VSMCs

Akt controls prosurvival and antiapoptotic activities. Upon activation Akt triggers several cellular effects including, cell proliferation, survival, cell growth, cell death, and migration by activating or inactivating several downstream target effector proteins. These proteins include FOXO transcription factors, mTOR, GSK3, and pro and antiapoptotic proteins in response to various cellular stimuli [47]. To determine whether butyrate-induced inactivation of Akt impacts its downstream target effector proteins in such a way that they mediate butyrate-induced inhibition of VSMC proliferation and cellular changes leading to cell growth, we examined the effect of butyrate treatment on FOXO transcription factors, caspase 3, PARP and mTOR.

#### 2.5.1. Impact of Butyrate-Inhibited Akt Activation on FOXO1 and FOXO3 Transcription Factors

**Figure 5:**
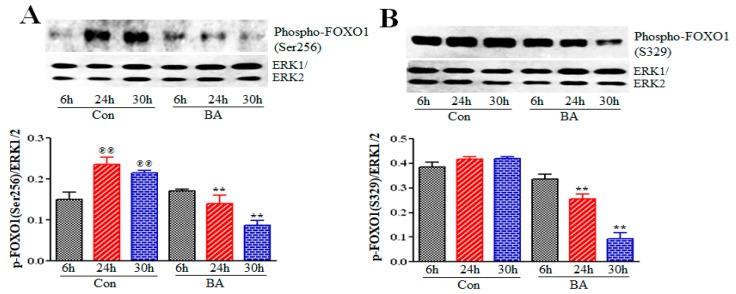
Evaluation of butyrate influence on phosphorylation state of FOXO1. VSMCs treated with (BA) or without (Con) butyrate for indicated periods were processed for Western blot analysis of phosphorylated FOXO1 at Ser256 and Ser329. (**A**) Phosphorylation state of FOXO1 at Ser256 is assessed by immunoblotting with antibody specific for FOXO1 phosphorylated at Ser256. (^℗℗^ *p* < 0.001 vs 6 h control) and is notably reduced in butyrate-treated VSMCs at 24 h and 30 h (** *p* < 0.001 vs respective control). (**B**) Phosphorylation of FOXO1 at Ser329 is determined by the antibody specific to FOXO1 phosphorylated at Ser329. (** *p* <0.001 vs respective control).

**Figure 6:**
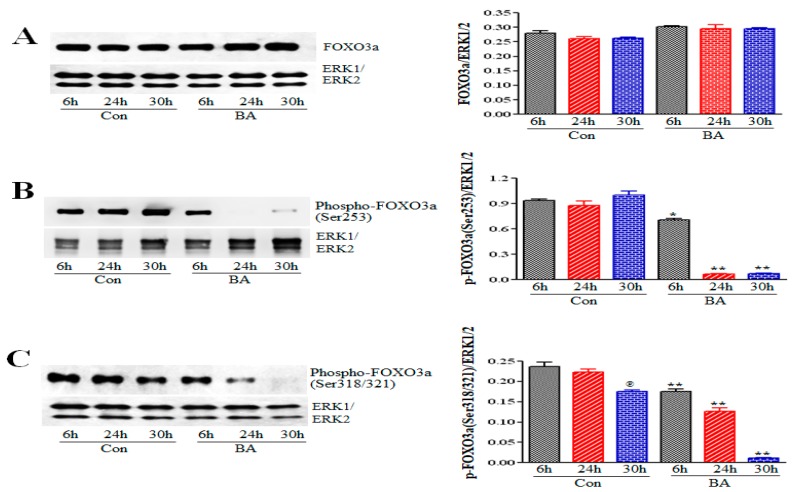
Assessment of butyrate effect on FOXO3. Cell lysates prepared from VSMCs treated with (BA) or without (Con) butyrate for indicated periods were subjected to Western blot analysis. (**A**) Analysis of total FOXO3 by immunoblotting with antibody specific to FOXO3. (**B**) Phosphorylation of FOXO3 at Ser253.* *p*< 0.01 vs 6 h control (Con); ** *p*< 0.001 vs respective control (Con). (**C**) Phosphorylation of FOXO3 at Ser318/321. ^℗^ *p*< 0.01 vs 6 h control (Con); ** *p*< 0.001 vs respective control (Con).

Active Akt supports survival of cells by blocking apoptosis through inhibition of function or expression of proapoptotic proteins and processes. FOXO proteins, a subgroup of Forkhead transcription factors, are direct targets of Akt, which regulate transcription of distinctly different groups of genes, depending on the cellular context to produce context-specific phenotypic effects. Primarily, Akt-mediated phosphorylation of FOXO proteins suppresses activation of FOXO proteins blocking transcription of genes linked to cell-cycle arrest or apoptosis [48,49,50]. Phosphorylation of FOXO proteins by Akt stimulates their association with 14-3-3 proteins, causing their translocation from the nucleus to the cytoplasm, thus preventing their transcriptional activity due to cytoplasmic sequestration. To examine the impact of butyrate-inhibited Akt activation on the transcriptional activity of FOXO proteins, the phosphorylation state of FOXO1 (Figure 5) and FOXO3, which is also referred to as FOXO3a (Figure 6) was analyzed by Western blot analysis. Butyrate treatment of VSMCs significantly reduced phosphorylation of FOXO1 at Ser256 (Figure 5A) and at Ser329 (Figure 5B) after 24 hours of treatment, compared to respective untreated VSMCs. Butyrate treatment also causes inhibition of phosphorylation of FOXO3 at Ser253 (Figure 6B) and at Ser318/321 (Figure 6C) compared to respective untreated controls. These results indicate that butyrate-induced inactivation of Akt leads to inhibition of phosphorylation of FOXO1 and FOXO3, suggesting that butyrate stimulates transcriptional activation of FOXO1 and FOXO3.

#### 2.5.2. Butyrate Effect on Caspase 3 and Poly (ADP-Ribose) Polymerase (PARP) in VSMCs

**Figure 7:**
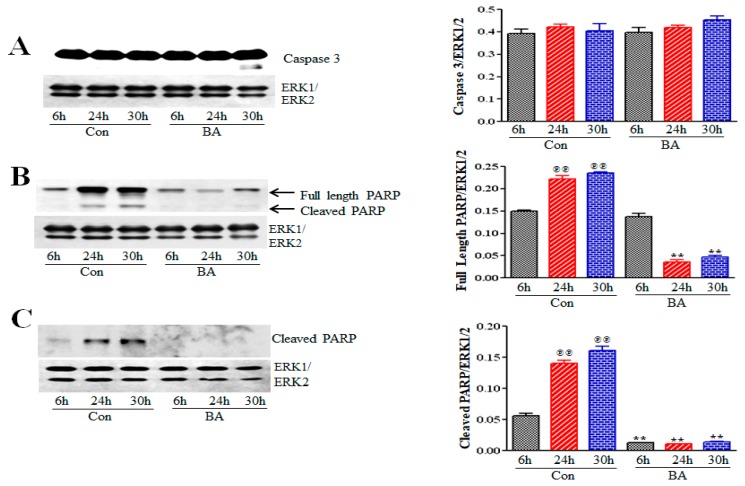
Evaluation of butyrate effect on caspase 3 and PARP in VSMCs. Cell lysates prepared from VSMCs treated with (BA) or without (Con) butyrate were processed for Western blot analyses using antibodies specific to respective proteins. (**A**) Caspase 3 exhibits no activation both in control and butyrate-treated VSMCs except for trace level of cleaved caspase 3 in VSMCs treated with butyrate for 30 h (**B**) Full length PARP was determined by immunoblotting with antibody specific to full length PARP (^@@^ *p* < 0.001 vs 6 h control and ** *p* < 0.001 vs respective control). (**C**) Cleaved PARP was assessed by probing with antibody specific to cleaved PARP (^@@^ *p* < 0.001 vs 6 h control but no cleaved PARP was detected in treated VSMCs (** *p* < 0.001 vs respective control).

Akt also participates in both promoting and inhibiting apoptosis by modulating expression of pro- and antiapoptotic proteins, including BAD, BAX, BAK, Bcl-2, CREB, MDM2, as well as modulating intrinsic mitochondrial pathway of apoptosis through its direct targets FOXO proteins and GSK3 [48,49,50,51,52]. Even though butyrate-induced changes in cellular morphology of VSMCs does not appear to indicate apoptosis [23,24,36,53], we still examined the effect of butyrate on the state of caspase 3 and PARP (Figure 7). The results indicate that the overall major portion of caspase 3 is intact in both untreated and butyrate-treated VSMCs except for trace level of cleaved caspase 3 in VSMCs treated with butyrate for 30 h (Figure 7A). Interestingly, while a time-dependent increase in intact and cleaved PARP is detected in untreated control VSMCs (Figure 7B,C), significant reduction in intact PARP and absence of cleaved PARP is observed in butyrate-treated VSMCs all through the treatment period (Figure 7B,C). These results support the notion that butyrate-induced cell-cycle arrest and cellular changes are not associated with apoptosis.

#### 2.5.3. Impact of Butyrate-Inhibited Akt Activation on Mammalian Target of Rapamycin (mTOR)

**Figure 8:**
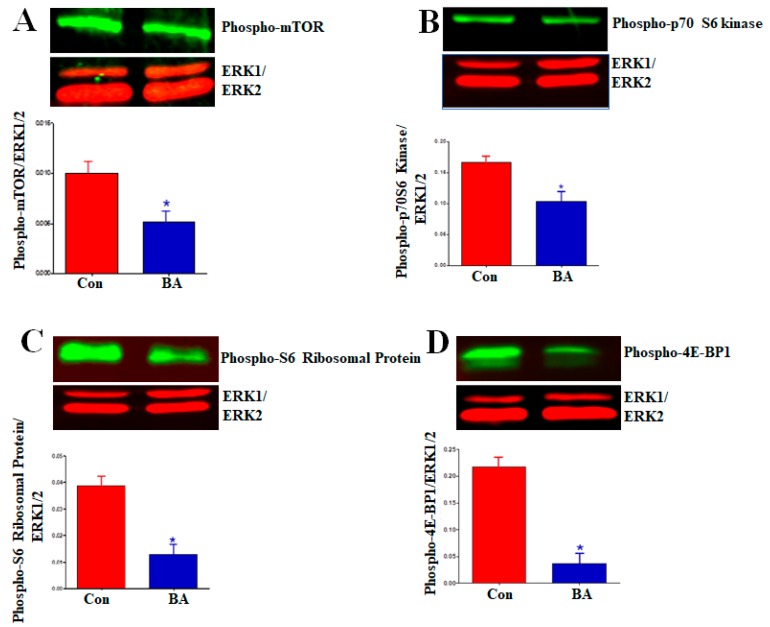
Inhibition of activation of mTOR and its downstream targets by butyrate in VSMCs. Cell lysates prepared from VSMCs treated with (BA) or without (Con) butyrate were analyzed by Western blotting using antibodies specific to (**A**) phosphorylated mTOR, (**B**) Phosphorylated p70S6 kinase, (**C**) phosphorylated S6 ribosomal protein and (**D**) phosphorylated 4E-BP1. Appropriate second antibodies labelled with IRDye 680LT or IRDye 800CW were used for detecting target proteins. LI-COR CLx imaging system and Image Studio software were used for scanning and quantitating signal intensities. (* *p* < 0.01 vs Control).

The serine/threonine kinase mTOR is the catalytic component of two distinct multiprotein signaling complexes, rapamycin-sensitive mTORC1 and rapamycin-insensitive mTORC2. As a part of the PI3K/Akt/mTOR pathway, mTOR exhibits a dual role as both an upstream activator of Akt and the downstream effector of the PI3K/Akt/mTOR pathway activity influencing cell growth, differentiation and metabolism by regulating protein synthesis and mRNA translation [47,54,55,56]. Activation of Akt by phosphorylation of serine residue Ser473 by mTORC2 and threonine residue Thr308 by PDK1 is required for mTORC1 activation. Since butyrate treatment of VSMCs causes cell growth through changes in cellular size and morphology, and the mTOR plays a role in cell growth by controlling protein synthesis, the consequence of butyrate-inhibited Akt activation on mTOR activation was examined in VSMCs treated with or without butyrate for 30 h (Figure 8A). Phosphorylation of serine residue, Ser2448 of mTOR, an indicator of activated mTOR [56], was assessed by Western blotting (Figure 8A). The results displayed in Figure 8A reveal butyrate treatment inhibits activation of mTOR by downregulating mTOR phosphorylation. This observation agrees with butyrate-inhibited Akt activation (Figure 4).

Upon phosphorylation-dependent activation, mTOR upregulates protein synthesis by exerting its kinase activity on its downstream target proteins p70S6 kinase and eukaryotic translation initiation factor-4E (eIF4E)-binding protein (4E-BP1), the key regulators of ribosome synthesis and mRNA translation [48,54]. Activated mTOR phosphorylates p70S6kinase, which in turn phosphorylates ribosomal S6 protein increasing general protein synthesis. Activated mTOR stimulates cap-dependent mRNA translation by phosphorylating 4E-BP1 and releasing it from inhibiting its binding partner, eIF4E. To confirm inhibition of mTOR activation in butyrate-treated VSMCs, the phosphorylation state of downstream targets of mTOR, including p70S6 kinase (Figure 8B), ribosomal S6 protein (Figure 8C) and 4E-BP1 (Figure 8D) were determined by Western blotting. The results displayed in Figure 8B–D corroborate with the attenuation of mTOR activation in butyrate-treated VSMCs. Furthermore, attenuation of mTOR and its downstream targets appears to indicate butyrate-induced cell growth is not mediated through mTOR.

## 3. Discussion

VSMCs play critical roles in atherosclerosis and restenosis-associated vascular remodeling leading to neointimal hyperplasia [2,3,37,38,39,57,58,59]. Their vulnerability to proliferate and migrate to the site of injury, to deposit extracellular matrix and secrete chemokines, cytokines and reactive oxygen species in response to injury leads to neointimal hyperplasia resulting in severe vascular events like heart attack, stroke and peripheral artery disease. Apoptosis of VSMCs is also a critical factor in atherosclerotic plaque-dependent cardiovascular events such as stroke, myocardial infarction and sudden death [2,49,60]. Loss of VSMCs via apoptosis provokes loss of structural integrity of the atherosclerotic fibrous cap that is susceptible to rupturing. Since cardiovascular disease continues to be a major health concern globally, any new interventional therapeutic development that reverses or prevents progression of atherosclerosis- and restenosis-associated remodeling in the vascular structural blueprint has significance in reducing the cardiovascular health menace. In this scenario, HDACIs appear to have potential use because of their effects on cell proliferation, survival and apoptosis. HDACIs have been highly implicated in the treatment of different cancers and other diseases [12,13,14,15,16] and recently in vascular diseases [2,4,22,37,38,39].

**Figure 9:**
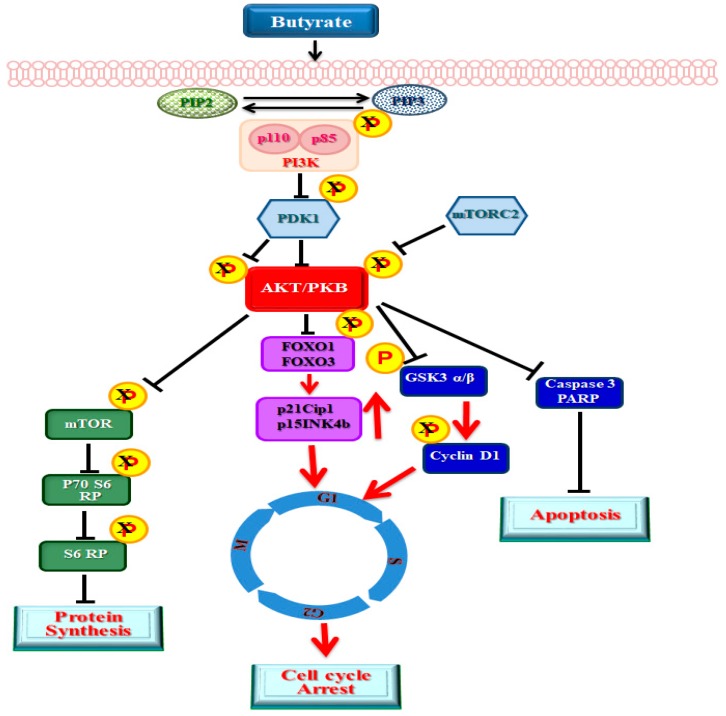
Butyrate-inhibited Akt activation arrests VSMC proliferation promoting VSMC growth via its downstream targets. Increased expression of cyclin D1, and its stability due to inhibition of GSK3 activity along with increased expression of p21Cip1/Waf1 and p15INK4B through the activation of FOXO proteins by butyrate arrests VSMCs in G1-phase (red arrows). Additionally, inhibition of caspase 3 activity and downregulation of PARP by butyrate inhibits apoptosis. Inhibition of mTOR-mediated protein synthesis appears to have no effect on VSMC growth.

Butyrate is a natural HDACI and a fermentation product of dietary fiber that potentially mediates the beneficial effects of dietary fiber. The physiological concentration of butyrate is about 10 to 20 mM based on the type of dietary fiber consumed. For most cell culture and animal studies, mM concentrations of butyrate are used ranging from 0.5 mM to 10 mM. In many studies 5 mM concentration appears to be effective concentration [29,33,37], including in our study [23]. Our earlier study used 0.5 mM to 8 mM butyrate to determine concentration-dependent effect of butyrate on VSMCs proliferation [23]. At 5 mM and higher than 5 mM up to 8 mM concentration, butyrate completely inhibits VSMC proliferation, but stimulates VSMC growth by increasing cellular size and mass, not by cell number. Furthermore, butyrate has been shown to elicit a multitude of cellular responses that support antiatherogenic effects from our previous studies [23,24,34,36,41] and studies from other investigators [37,38,39,40]. Our earlier studies also reveal that, in addition to inhibiting proliferation, butyrate also alters cellular size and morphology of VSMCs stimulating VSMC growth [23,24,36,53], and its effects are reversible upon its removal [34,53]. VSMCs react to butyrate treatment by gradually increasing their size and changing shape. Moreover, the scope of increase in size relies on the initial densities of the cultures [34]. Although the exact underlying mechanisms mediating these different cellular effects of butyrate are not clear, outcomes of the present study suggest butyrate effects on VSMCs are mediated through disparate effects on dual targets, HDAC activity and PI3K/Akt signaling network (Figure 9). HDAC activity that regulates gene expression by histone epigenetic modifications, causing chromatin remodeling [24,36], and PI3K/Akt signaling network that promotes activation or inactivation of certain effectors of PI3K/Akt pathway, are central to cell growth, proliferation, survival and apoptosis, respectively.

PI3Ks are a family of dual protein and lipid kinases, which upon activation phosphorylate the hydroxyl group at position 3 of the inositol ring of inositol phospholipids present in the plasma membrane. Class I PI3Ks are heterodimers of 85kDa regulatory subunit p85 and 110 kDa catalytic subunit p110, which are linked to an array of different functions. These functions include cell growth, proliferation, differentiation, survival and migration [44,45,46,47]. The PI3K/Akt signaling pathway, the pathway of Akt named after the upstream activator protein PI3K, plays a central role in several different cellular functions. These include cell proliferation, growth, survival, apoptosis, migration, differentiation, metabolism, and angiogenesis, which are important in cardiovascular function and structural integrity [44,45,46,47,48]. Assessment of this pathway in butyrate-induced cellular effects on VSMCs (Figure 1) discloses reduction in expression and inactivation of p85 regulatory subunit of PI3K, which is used as a measure of PI3K activity (Figure 2). Activity of PI3K is routinely assayed by determining the level of phosphorylated p85Tyr458 by Western blotting [61,62]. Furthermore, butyrate treatment also inhibits activity of PDK1, the upstream activator of Akt (Figure 3). Butyrate treatment of VSMCs inhibits phosphorylation of Ser241 of PDK1, which is required for its kinase activity. Therefore, it is not surprising that inhibition of PI3K and PDK1, the two upstream activators of Akt, attenuate Akt activation, as evidenced by inhibition of phosphorylation of both Thr308 and Ser473 residues of Akt that is central to Akt activation (Figure 4).

Our present study reveals increased phosphorylation of Thr308 and Ser473 residues of Akt in 6 h and 24 h untreated VSMCs, compared to corresponding butyrate-treated VSMCs. However, compared to 6 h untreated VSMCs, 24 h and 30 h untreated VSMCs exhibited significant reduction in phosphorylation of both residues of Akt. This inhibition of Akt activation in 24 h and 30 h untreated VSMCs is not unusual. Studies have shown that stimulation of cells with serum or growth factors, induces Akt activation [29,37,63,64,65]. This Akt activation, from very low to a period of increase in activation by phosphorylation, varies from minutes to hours to days, followed by reduction in Akt phosphorylation, depending on the cell type and cellular context [29,37,63,64,65]. Regarding appearance of slight reversal of butyrate inhibition of Thr308 and Ser473 phosphorylation in 30 h butyrate-treated VSMCs, it is possible that it may be linked to continuous incubation in the same culture medium for 30 h, without replenishing with fresh culture medium containing butyrate. Considering that butyrate effects are reversible upon removal of butyrate (34,53), it is possible that depletion or inactivation of butyrate in the culture medium may be linked to the state of Akt phosphorylation in 30 h butyrate-treated VSMCs. Despite these differences, butyrate treatment does inhibit Akt activation in VSMCs. Akt is the critical node of the PI3K/Akt pathway that is responsible for the regulation of different cellular processes of PI3K/Akt pathway through the mediation of serine and/or threonine phosphorylation of its downstream targets. Some of the important Akt targets that are essential to cardiovascular functions and structural integrity include FOXO transcription factors, mTOR and GSK3. Inactivation of Akt by butyrate differently affects FOXO transcription factors (Figure 5 and Figure 6), caspase 3 and PARP (Figure 7), mTOR (Figure 8), and GSK3 [36], ultimately promoting proliferation arrest and altered cellular size and morphology. This causes VSMC growth, possibly through appropriate collaborative cross-talk between the signal effectors of PI3K/Akt pathway (Figure 9).

Inhibition of cell proliferation by butyrate promotes different cellular effects including cell survival, cell growth, cell differentiation or cell death depending on the cell type and cellular context [24,28,29,30,31,32,33,36,37,40,41]. In serum-starved pulmonary arterial VSMCs (PAVSMCs), butyrate treatment inhibits platelet-derived growth factor (PDGF)-induced proliferation and migration [37]. In bovine kidney epithelial cells butyrate induces apoptosis and cell-cycle arrest [28]. Butyrate also inhibits growth of different tumor cells by inhibiting cell proliferation [30], promoting cell differentiation [33], inducing apoptosis [29] and preventing cell migration [31]. Unlike in these cells, our studies have shown that butyrate causes proliferation arrest and alters cellular morphology of VSMCs by a cytostatic effect, promoting cell growth [23,24,36,53]. Akt plays a crucial role in different cellular effects. Activation of Akt by phosphorylation of Ser473 and Thr308 residues is critical for promoting proliferation, growth and survival in a signal-context manner to extracellular signals or agents. Several studies reveal suppression of Akt activity is associated with the antiproliferative effect of butyrate resulting in inhibition of cell migration [31,37] and apoptosis [29]. The present study reveals suppression of Akt activation is associated with butyrate-induced VSMC growth by arresting VSMC proliferation (Figure 4). While these different butyrate-induced cellular effects are associated with Akt inactivation, it is the cellular environment and the ability of the cells to respond to their milieu that appears to be the determinant of their cellular fate through appropriate regulation of downstream targets of Akt [44,45,46,47,48].

Activated Akt elicits many cellular effects including cell survival, growth, proliferation, migration, metabolism and apoptosis that are important for cardiovascular functions and structural integrity through the mediation of serine and/or threonine phosphorylation of an assortment of downstream targets. Phosphorylation by Akt can be inhibitory or stimulatory, thus, negatively or positively suppressing or enhancing the activity of target proteins. Moreover, some crucial Akt targets including FOXO transcription factors, mTOR and GSK3, control more than one cellular function and their role may vary in a cell and signal-context dependent manner. As such, FOXO transcription factors, mTOR and GSK3 control several cellular processes that include cell survival, growth, proliferation, metabolism, and apoptosis. The role of these Akt targets in butyrate-induced cellular effects are not well understood, particularly in butyrate-inhibited VSMC proliferation and altered cellular morphology. Our present study reveals abrogation of Akt activation by butyrate differentially affects FOXO1 and FOXO3 transcription factors, mTOR, caspase 3 and PARP, and GSK3 [36]. Convergence of cross-talk between their actions apparently leads to proliferation arrest and altered morphology of VSMCs leading to cell growth.

FOXO1 and FOXO3 proteins, members of a subgroup of Forkhead transcription factors, play pivotal roles both in cell-cycle arrest, and/or apoptosis signaling by coordinating a cast of gene expression that regulate cell survival and cell death [48,49,50]. Activated Akt directly phosphorylates FOXO proteins impairing their DNA-binding activity but boosting their affinity for 14-3-3 chaperone proteins, thus, facilitating export of FOXO-14-3-3 complex out of the nucleus to the cytoplasm. Cytoplasmic sequestration of FOXO proteins blocks FOXO-dependent transcriptional activation. Conversely, inactivation of Akt triggers transcriptional activation of FOXO proteins by promoting their residence in the nucleus, eliciting expression of multiple target genes specific to cell-cycle arrest or apoptosis depending on the cell type and cellular context [49,50,66]. Moreover, studies have shown that FOXO proteins enhance cell-cycle arrest by upregulating the levels of different cyclin-dependent kinase (cdk) inhibitors that belong to the INK and Cip/Kip family as an essential part of the cytostatic response by stimulating G1 arrest resulting in quiescence [24,67]. In the present study, inhibition of FOXO1 (Figure 5) and FOXO3 (Figure 6) phosphorylation in butyrate-arrested VSMC proliferation indicates transcriptional activation of FOXO proteins, especially those genes that promote cell-cycle arrest. In concurrence with this possibility, our earlier studies have shown butyrate treatment of VSMCs induces cell-cycle arrest and promotes entry to quiescence with no obvious signs of cell death, along with upregulation of p21Cip1/Waf1 and p15INK4B [24,36]. Correspondingly, several other studies also demonstrate FOXO proteins play a major role in cell-cycle arrest by enhancing cdk inhibitors, p21Cip1/ Waf1 and p15INK4 levels [67]. Recent studies indicate that FOXO activity is controlled by an intricate combination of post-translational modifications (PMTs) including acetylation, methylation and ubiquitination, in addition to phosphorylation [68]. These modifications in turn regulate their subcellular localization, expression level and DNA-binding activity. Considering that butyrate is an inhibitor of Class I and Class II HDACs, it will be interesting to explore butyrate’s influence on acetylation of FOXO proteins enhancing their transcriptional activation. Additional studies are required to explore the impact of butyrate on transcriptional activation of FOXO proteins that contributes to the cytostatic response [24,36].

A range of unrelated factors and conditions, including chemotherapeutic agents, loss of contact with the extracellular matrix, DNA damage, and growth factor withdrawal can initiate the intrinsic mitochondrial pathway of apoptosis, resulting in the activation of caspase cascade. Although several studies reveal that butyrate arrests cell proliferation by inducing apoptosis [22,28,29], our studies reveal that butyrate exerts a cytostatic response in VSMCs with no obvious signs of apoptosis [24,36,53]. To substantiate this observation, we examined butyrate’s effect on the activity state of caspase 3 and PARP, representing caspase-dependent and caspase-independent mechanisms of apoptosis, respectively (Figure 7). As expected, there was no overall change in the levels of intact caspase 3 in both untreated and butyrate-treated cells, with the exception that sometimes a minimal cleavage of caspase 3 in VSMCs treated for 30 h with butyrate was noticed (Figure 7A).

Interestingly, in butyrate-treated VSMCs, intact uncleaved PARP level was not only significantly downregulated, but there was also no cleavage of PARP, compared to untreated VSMCs (Figure 7B,C). Furthermore, significant increase in the levels of cleaved PARP fragment in both 24 h and 30 h untreated VSMC cultures is surprising. It is possible that the presence of cleaved PARP in untreated VSMCs may be linked to disintegration of cells that may have lost contact inhibition once the culture becomes confluent. Although butyrate effects on caspase 3 and PARP further support that apoptosis is not involved in butyrate-inhibited VSMC proliferation, the effects of butyrate on PARP is puzzling, and at the same time exciting, because it may have therapeutic implications. PARP was initially identified as the enzyme that detects and initiates repair of single-, double-stranded DNA breaks. Recent studies recognize that it is a multifunctional enzyme, and its functions depend on its extent of activation [69,70]. Besides its role in DNA-damage repair, it is a well-known apoptotic marker, and its hyperactivation leads to accumulation of poly-ADP-ribose (PAR) and depletion of NAD^+^ and ATP, causing caspase-independent apoptosis or necrotic cell death. Unwarranted hyperactivity of PARP has been alluded to the pathogenesis of several diseases and disorders, including stroke, myocardial infarction, inflammation, diabetes, cancer and neurodegenerative disorders [69,70]. In this scenario, butyrate-induced downregulation of PARP appears to strengthen butyrate’s therapeutic potential in other diseases besides its use as an anti-cancer agent. However, further clarification is required to understand how butyrate regulates PARP.

Besides arresting proliferation and causing no cell death, butyrate alters cellular morphology of VSMCs from their elongated bipolar morphology to a round and spread-out phenotype with increase in sizes, implicating inhibition of cell proliferation by butyrate promotes cell growth [23,24,36,53]. Our studies also observed the extent of size increase is dependent on the initial densities of the cultures, and it is due to increase in mass, not due to increase in cell number. Recent studies indicate, increase in cell size due to increase in growth is possible through two mechanisms: a cell-cycle-related mechanism through an increase in cyclin D1 level that prolongs the stay of the cells in G1-phase of the cell cycle, and the mTORC1 signal pathway-mediated mechanism that boosts protein synthesis [71,72,73,74]. According to our previous studies, a cell-cycle related mechanism is linked to butyrate-induced cellular growth [24,36]. In response to mitogenic signals, quiescent cells enter into G1-phase of the cell cycle, increase cyclin D1 level, activate cyclin D1/cdk4 activity and stimulate protein synthesis prior to transition to S-phase. However, in the presence of antiproliferative agents, cell-cycle progression is arrested before the restriction point. This leads to inhibition of cyclin E/cdk2 activation and attenuation of the movement of cells into S-phase. Consequently, it promotes increase in physical growth in early G1-phase without progressing entry into S-phase [75,76]. Moreover, increased expression of the INK and Cip/Kip families of cdk inhibitor complexes also contributes to cell-cycle arrest triggering cell growth [24,36,77,78]. Correspondingly, our earlier studies demonstrate butyrate-induced cellular changes of VSMCs were linked to butyrate effects on cell-cycle regulatory proteins. Butyrate not only increases cyclin D1 level that is localized to nuclear regions, it also induces expression of cyclin D type-specific cdk4/cdk6 inhibitor p15INK4B and universal cdk inhibitor p21Cip1/Waf1 that inhibit G1/S-phase cdk complexes [24,36]. In general, most HDACIs arrest cell proliferation in G1-phase with universal increased expression of p21Cip1/Waf1. Taken together, butyrate-induced enhanced levels of both cyclin D1-specific cdk inhibitor p15INK4B and universal cdk inhibitor p21Cip1, and augmented levels of cyclin D1 itself, force VSMCs to stay in G1-phase and grow in size, preventing proliferation by restraining them from progressing into S-phase.

Our previous studies also reveal that increase in cyclin D1 levels is linked to increased synthesis and increased stability, based on cyclin D1 transcription and phosphorylation state of GSK3, respectively [36]. GSK3 is an important downstream target of Akt that exists in two isoforms α and β, regulating several cellular functions, including cell growth, survival, death, proliferation, and metabolism, by its role in various signaling pathways that control the fate of the cell [49]. Activation of Akt triggers inhibitory phosphorylation of GSK3, causing its inactivation via phosphorylating Ser9 on GSK3α and Ser21on GSK3β, which permits cell proliferation [49,51,52]. Conversely, inhibition of Akt activation in response to certain stimuli can cause cell-cycle arrest by activating GSK3 kinase activity through dephosphorylation of GSK3 [36,52]. Activated GSK3 functions in cell-cycle regulation by phosphorylating Thr286 on cyclin D1 that plays a crucial role in G1 to S-phase cell-cycle transition. Although our earlier studies indicate GSK3 is phosphorylated and inactive in response to butyrate treatment, the upstream signaling pathway linked to GSK3 inhibition in butyrate-arrested VSMC proliferation was not investigated [36]. Interestingly, the present study reveals that inactivation of Akt, the usual regulator of GSK3, by butyrate (Figure 4) was not linked to inactivation of GSK3 [36]. It is possible protein kinases of other signaling pathways may be involved in the inhibitory phosphorylation of GSK3 that stabilizes cyclin D1, by significantly inhibiting Thr286 phosphorylation-dependent degradation of cyclin D1, thereby resulting in nuclear accumulation of cyclin D1 in G1-phase appears to be responsible for cellular changes, and arrest of VSMCs in G1-phase.

Induction of protein synthesis in G1-phase is required for cell growth, and it is acknowledged that mTOR signaling pathway and its downstream targets, p70 ribosomal S6 kinase (p70S6K) and eukaryotic translation initiation factor 4E binding protein (4EBP1) dependent signals are key to regulating growth by increasing protein synthesis [47,54,71,72,73,74]. Our present study reveals, mTOR is not required for the butyrate-induced increase in size/growth of VSMCs. Treatment of VSMCs with butyrate inhibits phosphorylation of mTOR, which inevitably leads to inhibition of phosphorylation of downstream effectors of mTOR, p70S6 kinase and 4EBP1 (Figure 8). Considering the inactivation of Akt, the upstream activator of mTOR pathway by butyrate, it is not surprising that mTOR pathway is inactivated. These observations indicate, butyrate-induced increase in VSMC growth may involve mTOR-independent pathways that control protein synthesis in VSMC growth. Similar mTOR- independent regulation of protein synthesis, and cell growth have been reported in some cases [72,74]. The reasons for disparity are not clear, but perhaps, different cell types have different degrees of necessity for mTOR in control of their growth or probably alternate compensatory pathways are upregulated. In this regard, cyclin D1 itself exhibits several cdk-independent functions, causing distinct cellular effects through its interaction with more than 30 different transcription factors and transcription regulators, including HDACs and HATs, which may contribute to VSMC growth [79].

## 4. Materials and Methods

### 4.1. Cell Culture and Treatment of VSMCs

Rat aortic VSMCs obtained from commercial source (Cell Applications Inc., San Diego, CA, USA) were used for studies. They were cultured and maintained in Dulbecco’s modified Eagle’s medium (DMEM) augmented with 10% fetal bovine serum, 100 Units of penicillin and 100 µg/ml streptomycin (Atlanta Biologicals, Lawrenceville, GA), used as complete medium, in a humidified atmosphere of 5% CO2 at 37 °C. Confluent cultures were subcultured by seeding the cells at a ratio of 1:4. One day after seeding, proliferating cells were untreated or treated with 5 mM butyrate (Sigma-Aldrich Chemical St. Louis, MO, USA) for indicated periods of time as described previously [24,36]. For all studies, 5 mM butyrate concentration was used based on our previous concentration-dependent studies [23,75]. Butyrate concentrations ranging from 0.5 mM to 8 mM revealed, 5 mM and higher than 5 mM up to 8 mM concentrations, completely inhibited VSMC proliferation causing no cytotoxicity or apoptosis [23,75]. Cultures were fed with fresh complete medium containing butyrate or no butyrate every other day unless otherwise mentioned. At the end of incubation period, VSMCs were collected for analysis after washing with phosphate-buffered saline (PBS). Unless mentioned, results presented are derived from two to three independent experiments. For all studies, third to tenth passage of VSMCs was used.

### 4.2. Butyrate-Induced Cellular Effects on VSMCs

To determine the effect of butyrate on VSMC growth, one day after seeding, proliferating VSMCs were treated with complete medium containing butyrate (BA) or no butyrate (Con) for 72 h. After 72 h of treatment, cultures were washed with PBS and fixed in cold methanol for 5 minutes as described previously, to assess butyrate-induced altered cellular morphology and increase in size that promotes VSMC growth [24,36,41]. After fixing, washed with PBS three times, then blocked with 10% heat inactivated horse serum (HS) in PBS for one hour at room temperature. After washing with PBS, cultures were incubated with 1µg/ml Hoechst in 1.5% HS for 30 min. After incubation, cultures were washed with PBS and used for fluorescence microscopy using a Nikon fluorescence microscope.

To demonstrate butyrate-induced VSMC growth is linked to proliferation arrest, VSMC cultures exposed to butyrate for 72 h, as described above. After 72 h of treatment, cultures were washed three times with sterile PBS and trypsinized with trypsin-EDTA. Cells were collected in DMEM and centrifuged briefly at 3000 rpm at room temperature. The cell pellets were suspended in PBS and cell numbers were counted by trypan blue exclusion method, using TC20 Automated cell counter from Bio-Rad, as described by the manufacturer (Bio-Rad, Hercules, CA, USA).

### 4.3. Western Blot Analysis

For Western blot analysis, one day after seeding the cells, cultures were treated with complete medium containing (BA) or no butyrate (Con) for indicated periods without replacing with fresh culture medium. At the end of treatment period, cultures were washed with ice cold PBS. Total cell lysates from experimental cultures were prepared and quantitated for protein concentration as described previously [41]. Briefly, total cell lysates from experimental cultures were prepared by lysing the cells in sodium dodecyl sulfate (SDS) sample buffer containing protease and phosphatase inhibitors (5872, Cell Signaling Technology, Danvers, MA, USA) by gentle rocking for 10 min at 4 °C. The lysates were collected, and homogenized by passing through syringe and 21 gauge needle 15 times [41]. The homogenates were centrifuged at 15,000 rpm for 15 min at 4 °C. The supernatants were collected and protein concentrations were determined with the BCA protein assay kit from Pierce Biotechnology (Rockford IL, USA). Required amounts of samples were denatured by heating at 90 °C for 5 min, after adding β-mercaptoethanol and SDS to a final concentration of 1% and 0.05%, respectively. Equal amounts of protein from each sample were subjected to sodium dodecyl sulfate-polyacrylamide gel electrophoresis (SDS-PAGE) and separated proteins were electro-blotted onto PVDF membrane and used for Western blotting.

Western blotting was performed first blocking the blots with Tris-buffered saline (TBS) containing 0.1% Tween 20 and 5% non-fat milk powder (Blotto sc-2324, Santa Cruz Biotechnology, Dallas TX, USA) for 1 h at room temperature. Then the blots were incubated overnight at 4 °C with appropriate primary antibodies to detect activation and/or levels of target proteins with gentle agitation [24,36,41]. The primary antibodies that were used include p85PI3Kinase (4257, 1:1000 dilution), phospho-p85PI3Kinase (4228, 1:1000 dilution), phospho-Ser241PDK1 (3438, 1:1000 dilution), PDK1 (3062, 1:000 dilution) Akt1 (4691, 1:1000 dilution), phospho-Ser473Akt (4060, 1:1000 dilution), phospho-Thr308Akt (2965, 1:1000 dilution), phospho-Ser2448mTOR (5536, 1:1000 dilution), phospho-Thr389p70S6Kinase, (9234, 1:1000 dilution), phospho-Ser235/236-S6 ribosomal protein (4858, 1:1000 dilution), phospho-Thr37/46–4E-BP1 (2855, 1:1000 dilution), phospho-Ser318/321FOXO3a, (9465, 1:1000 dilution), phospho-Ser256FOXO1, (9461, 1:1000 dilution), cleaved caspase 3 (9661, 1:1000 dilution), full length PARP (9532, 1:1000 dilution), cleaved PARP (9545, 1:1000 dilution), ERK1/ERK2 (9102, anti-rabbit, 1:1000 dilution) and ERK1/ERK2 (9107, mouse monoclonal, 1:2000 dilution) from Cell Signaling Technology (Danvers, MA, USA). FOXO3a (GTX62705, 1:2000 dilution) was from GeneTex (Irvin, CA, USA), Phospho-Ser329FOXO1 (ab52857, 1:2000 dilution) and phospho-Ser253FOXO3a (ab154786, 1:2000 dilution) from abcam (Cambridge, MA, USA).

Following primary antibody incubation, the blots were incubated with species appropriate horse radish peroxidase (HRP) conjugated anti-rabbit-HRP-linked secondary antibody (7074, 1:2000 dilution) from Cell Signaling Technology (Danvers, MA, USA) or IRDye-conjugated goat anti-mouse IRDye 680LT (925-68020, 1:20,000 dilution) and goat anti-rabbit IRDye 800CW (925-32211, 1:15,000 dilution) secondary antibodies from LI-COR, Inc. (Lincoln, NE, USA). Blots probed with HRP-conjugated secondary antibodies were processed for chemiluminescence immunodetection using luminol reagent from Santa Cruz Biotechnology (Dallas, TX, USA). Band intensities were measured and analyzed by using Molecular Imager Pro Plus MultiImager system and Quantity One software from Bio-Rad (Hercules, CA, USA). Blots that were probed with IRDye-conjugated secondary antibodies were imaged on Odyssey CLx imager and band intensities were measured and quantitative analysis was performed using Image Studio software from LI-COR, Inc. (Lincoln, NE, USA). For normalizing protein loading, blots were probed with anti-ERK1/ERK2 antibodies, which is unaffected in response to butyrate treatment [24,36,41] unlike routinely used normalizing controls such as GAPDH and β-actin. For some phosphorylated signal transduction pathway proteins, the membranes were probed with antibodies specific to their respective total protein to normalize the protein loading.

### 4.4. Data Analysis

Statistical analysis was performed using GraphPad Prism version 5 software from GraphPad Software Inc (La Jolla, CA, USA). Statistical differences between controls and treatment groups were analyzed by one-way analysis of variance (ANOVA) with Bonferroni multiple comparison test. Statistically significant differences between data sets were calculated at *p* < 0.01 and *p* < 0.001.

## 5. Conclusions

In conclusion, butyrate’s cellular effects on VSMCs are mediated through disparate effects on dual targets, HDAC activity and PI3k/Akt signaling network. Butyrate arrests VSMC proliferation and inhibits cell death, promoting cell growth by increasing cellular size. Through its HDAC inhibitory functionality, butyrate not only alters expression of epigenetically regulated genes associated with cell-cycle regulation and PI3K/Akt signaling network, but also interferes with PI3K/Akt signal transduction pathway through PTMs of Akt and Akt targets. Increased expression of cyclin DI and D3, p15INK4, p21Cip1/Waf1 [24,36] and clusterin (our unpublished data) in butyrate-treated VSMCs indicate multiple proteins are associated with change in VSMC phenotype. Considering cardiovascular disease continues to be a major health concern globally, any new interventional therapeutic development that reverses or prevents progression of atherosclerosis- and restenosis-associated remodeling of vascular structural blueprint has significance in reducing cardiovascular health menace. In this scenario, HDACIs, particularly the dietary HDACI butyrate, exhibits chemopreventive and chemotherapeutic potential, which have been already dominated in the treatment of different cancers and other diseases. Besides, butyrate-induced downregulation of PARP appears to strengthen butyrate’s therapeutic potential. Unwarranted hyperactivity of PARP has been linked to pathogenesis of several diseases and disorders including stroke, myocardial infarction, inflammation, diabetes, cancer and neurodegenerative disorders [64,65]. However, further clarification is required to understand how butyrate regulates PARP and its significance in vascular remodeling.
